# Correlation of Paraoxonase-1 with glycated hemoglobin and lipid profile among Sudanese diabetic patients

**DOI:** 10.12669/pjms.35.4.26

**Published:** 2019

**Authors:** Ahmed M Ahmed

**Affiliations:** Dr. Ahmed Mohammed Ahmed, Assistant Professor, Department of Clinical Laboratory Sciences, Faculty of Applied Medical Sciences, Taibah University, Al Madinah, Kingdom of Saudi Arabia

**Keywords:** Paraoxonase 1, PON1, T1DM and T2DM

## Abstract

**Objective:**

To examine concentration of Paraoxonase 1 enzymes across both Sudanese patients suffering from Type-I and Type-II diabetes.

**Methods:**

This was a cross-sectional study done in Khartoum/Sudan during the period from June 24th 2018 to August 23, 2018. One hundred seven diabetic patients (40 T1DM and 67 T2DM) compared with 45 healthy people from both genders. Biochemical parameters include PON1, FBG, HbA1C, and lipids were done and compared between study groups.

**Results:**

PON1 was reduced in patients than controls (P < 0.01), in addition PON1 was lower in T1DM than T2DM (P < 0.01), moreover, FBG, HbA1c and lipids was higher in diabetes than controls (P < 0.05). PON1 inversely correlated with LDL and apo B in T1DM (P < 0.01) and T2DM (P < 0.05), in addition PON1 correlated with HDL and apo A1 in T1DM (P < 0.01), inversely correlated with LDL in T2DM (P < 0.05) and with apo A1 in T2DM (P < 0.01). Moreover, PON1 inversely correlated with diabetes duration in T1DM (P < 0.01) and T2DM (P < 0.05).

**Conclusion:**

Sudanese T1DM and T2DM have a lower PON1 concentration than healthy subjects, T1DM have lower level of PON1 than T2DM. PON1 was inversely correlated with bad lipids and duration of diabetes, but it has positive correlation with good lipids.

## INTRODUCTION

Paraoxonase 1 (PON1) is a 44 kD Ca^2+^-dependent glycoprotein synthesized by the liver and associated with HDL. PON1 decreases lipid peroxide accumulation in LDL due to its antioxidant ability against hydroperoxides thus reduce LDL and phospholipid oxidation, Thus, PON1 may be involved against atherosclerosis protection.^1^ Diabetes mellitus (DM), is a life-threatening chronic disease associated with about 10% death episodes in adults.^2^ DM is leading risk factor for development of coronary heart diseases and mortality if not properly managed.^3^

In recent meta-analysis study that involved 35 studies worldwide, PON1 has been shown to decrease in DM and to be associated with the risk of diabetic macroangiopathy and microangiopathy.^4^ In addition, in another meta-analysis, PON1 polymorphisms have been shown to play an important role in the susceptibility of diabetic macroangiopathy and microangiopathy.^5^

The prevalence of diabetes in Sudan was high and increased from 3.4% in 2001^6^ to 10.9% in 2017.^7^ No published data found about PON1 in Sudanese diabetes, so the current research focus on evaluation of PON1 concentration status in Sudanese diabetic patients compared to healthy subjects.

## METHODS

In this cross-sectional study, 107 diabetic patients (mean age = 45±4.8) (40 T1DM and 67 T2DM) (65 male) were enrolled in study from Jabir Abu Aliz Diabetes Centre (Khartoum, Sudan) during the period June 24th 2018 to August 23, 2018. In addition, 45 healthy subjects (28 males) as a control group were included for comparison. Inclusion criteria for diabetes group: HbA1c more than 6.5%, random blood glucose (FBS) ≥ 7.7 mmol/l. Exclusion criteria included kidney failure, chronic illness like cancerous patients, liver diseases, anemias and thyroid disease. The permission of study was taken by Research Ethics Committee - Applied Medical Sciences - Taibah University, Madinah, Saudi Arabia. In addition, ethical approval was taken from Khartoum State - Ministry of Health - Research Ethics Committee – Khartoum – Sudan. Both committees followed the ethical standards of the 1964 Declaration of Helsinki and its amendments.^8^ Full description of study objectives was provided to all participants prior to obtaining of written informed signed consent.

### Anthropometric Parameters

Body mass index (BMI) was measured as (kg/m^2^) according to Quetelet equation.^9^ BMI categories were normal was (18.5-24.9 kg/m^2^), overweight was (25–29.9 kg/m^2^) and obese was (≥30.0 kg/m^2^).

### Laboratory Measurements

Blood samples (3 ml) were collected from all participants in plain tubes after 12-hour overnight fasting and serum was obtained using standard centrifugation method. FBG, total cholesterol, triglyceride, HDL, LDL, apolipoprotein A1 (apo A1) and apolipoprotein B (apo B) were measured by using an auto-analyzer (Hitachi 704 Roche Diagnostics Switzerland). HbA1c measured by D-10™ Hemoglobin Analyzer Bio-Rad (Nyocard), PON1 concentration was measured with Elabscience’s ELISA kit (Sandwich-ELISA principle) using fresh serum. According to protocol, 100 µl of samples and standard were added to ELISA wells (pre-coated microplate PON1) and incubated for 90 minutes at room temperature (RT), then biotinylated (detection antibody for PON1 specifically) was added and incubated for one hour in RT. After wash (3 cycles), avidin-horseradish peroxidase (HRP) was added as secondary antibody and incubated for 30 minutes at RT. The plates were washed 5 times and the substrate reagent was then added to wells and incubated for 15 minutes at RT. The reaction was terminated with 50 µl of Sulphuric acid solution and plates were read at wavelength 450 nm using ELISA reader. The concentration of PON1 was calculated by plotting optical density on the standard curve.

**Fig. 1:**
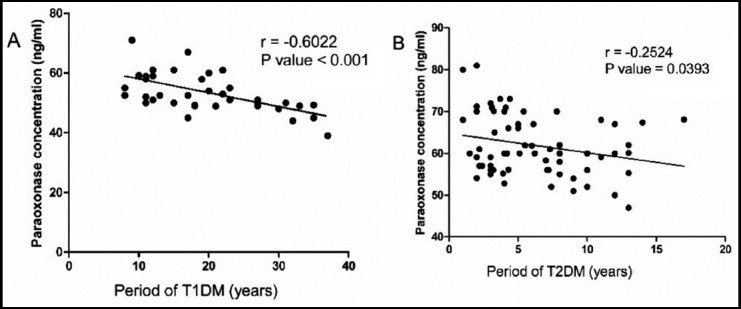
Show the correlation between PON1 and duration of diabetes, [A] express that PON1 was inversely correlated with the period in T1DM (r = -0.6022, P < 0.01) and [B] show PON1 was inversely correlated with the period in T2DM (r = -0.2524, P < 0.05).

### Statistical Analysis

The data were analyzed using GraphPad Prism version five statistical program (GraphPad Software, San Diego, CA, USA). Results represent as mean±SD. ANOVA (Tukey post hoc test for comparing 3 groups), t-test (for comparing 2 groups), chi-square (for non-parametric), and Pearson correlation were applied when needed. The scatter diagram designed with the GraphPad. P value ≤ 0.05 considered significant.

## RESULTS

Demographics (age, gender and BMI) and biochemical data are shown in Table-I. The mean age of T1DM, T2DM and controls were 45±2.7, 46±3.7, and 45±1.8 respectively (P = 0.126). Gender ratios across participants were not different (P = 0.533). BMI was higher in T2DM compared to T1DM and controls (P < 0.001). In addition, period of diabetes was higher in T1DM than T2DM (P < 0.001). Biochemical parameters including FBG, HbA1c, LDL and apo B were significantly elevated in patients (T1DM and T2DM) than control group (P < 0.001) [for Total cholesterol and Triglyceride (P < 0.05)]. Moreover, HDL apo A1 were significantly lower in patients T1DM and T2DM than control group (P < 0.001). With respect to Paraoxonase 1 enzyme level, it was lower in T1DM than T2DM and controls (P < 0.01).

**Table I:** Represents demographics and biochemical parameters of T1DM, T2DM and control subjects.

	T1DM (n= 40)	T2DM (n= 67)	Controls (n= 45)	P value
Age (years)	45±2.7	46±3.7	45±1.8	0.126 ^a^
Gender				
-Male	27	38	28	0.533 ^b^
-Female	13	29	17	
BMI	22±3.7	27±3.1*	23±2.4	<0.001 ^a^
Period of diabetes (year)	19.3±4.2	6.3±3.8	-	<0.001 ^c^
Glucose (mmol/l)	11.1±1.3	9.2±2.7	5.7±1.1	<0.001 ^a^
HbA1c %	9.2±2.2	8.6±3.1	5.4±0.8	<0.001 ^a^
Total Cholesterol (mmol/l)	4.18±0.9	4.0±0.8	3.71±0.3	0.01 ^a^
Triglyceride (mmol/l)	1.51±0.3	1.38±0.3	1.31±0.4	0.02 ^a^
LDL-Cholesterol (mmol/l)	4.3±0.8	4.1±0.7	2.6±0.2	<0.001 ^a^
HDL-Cholesterol (mmol/l)	1.1±0.5	1.2±0.5	1.9±0.1	<0.001 ^a^
Apolipoprotein A1 (g/l)	1.0±0.6	1.1±0.7	1.63±0.2	<0.001 ^a^
Apolipoprotein B (g/l)	13.2±4.3	12.8±3.7	9.2±2.2	<0.001 ^a^
Paraoxonase 1 (ng/ml)	53.7±11.8*	61.8±9.5	98.4±10.8	<0.001 ^a^

Data represent as (mean±sd).

a : ANOVA p value.

b : chi-square p value.

c t-test p value.

* Significant compare to controls & T2DM (p≤0.01), (t-test).

Table-II show Pearson correlation between PON1 concentration with HDL, LDL, apo A1, and apo B. PON1 was negatively correlated with LDL in both types of patients: T1DM (r = -0.5733, P < 0.01), T2DM (r = -0.4639, P < 0.05). Similarly, PON1 level was also significant negatively correlated with apo B in both patients type, T1DM (r = -0.6386, P < 0.01), T2DM (r = -0.4940, P < 0.05). On the other hand; the level of enzyme was significant correlated with HDL in both types, T1DM (r = 0.6957, P < 0.01), T2DM (r = 0.5116, P < 0.05). Also significant positively correlated with apo A1 in both types, T1DM (r = 0.7001, P < 0.01), T2DM (r = 0.5546, P < 0.01).

**Table II:** show Pearson correlation between PON1 enzyme level and LDL, HDL, apoA1 and apo B in T1DM & T2DM.

Correlations	r	P value	95% (CI)
PON1 & LDL			
-T1DM	-0.5733	0.0066	-0.8057 to -0.1881
-T2DM	-0.4639	0.0342	-0.7462 to -0.04012
PON1 & HDL			
-T1DM	0.6957	0.0005	0.3773 to 0.8670
-T2DM	0.5116	0.0177	0.1025 to 0.7727
PON1 & apo B			
-T1DM	-0.6386	0.0018	-0.8390 to -0.2856
-T2DM	-0.4940	0.0228	-0.7630 to -0.07911
PON1 & apo A1			
-T1DM	0.7001	0.0004	0.3845 to 0.8691
-T2DM	0.5546	0.0091	0.1615 to 0.7958

## DISCUSSION

The most important finding in the current study was the significant decrease of the concentration of PON1 enzyme in both T1DM and T2DM compared to healthy people. This is in agreement with a previous study from Czech in both T1DM and T2DM,^10^ and studies conducted on T2DM in different countries.^11^-^20^ In addition, the result is consistent with a previous report that examined PON1 levels in children with T1DM fromTunisia.^21^

Prolong hyperglycemia in patients with diabetes due to impairment of insulin pathway leads to oxidative stress (atherogenesis) and the subsequent induction of antioxidant mechanisms as a protective measure. PON1 plays a vital role as antioxidant enzyme, and has important role against oxidative damage. In DM, hyperglycemia causes glycosylation of proteins including enzymes and this process may lead to decrease their activity.^22^ In the current study, the concentration of PON1 enzyme was lower in T1DM than T2DM. This may be because the duration of diabetes in T1DM is usually longer than T2DM (P < 0.001) and the complete absence of insulin that leading to prolong glycation of the enzyme protein.

With respect to other biochemical parameters, glucose, HbA1c, total cholesterol, triglyceride, LDL and apo B they were significantly higher in T1DM & T2DM than controls (P < 0.001). This is in agreement with Indian T2DM,^16^ and Egyptian T2DM study.^17^ In a previous Czech study conducted on T1DM and T2DM, the results showed increased total cholesterol and triglyceride in T1DM than controls, but in T2DM, only triglyceride was significant increased.^10^ The current results also agree with a previous study conducted in Slovakia except the apo B that was different between T2DM and controls.^12^

The results showed that HDL and apo A1 were decreased in both diabetic groups than control subjects (P < 0.001). This is in agreement with the Slovakian T2DM study^12^ and disagree with Czech T1DM and T2DM^10^ and Indian T2DM^16^ study. The results are also in agreement with Egyptian T2DM (agree with HDL result but no apo A1 done).^17^ The differences between different studies in biochemical measures may be due to different ethnicity, various genetic types, environment and adherence to regiments.

It is well known that PON1 enzyme decreases accumulation of oxidized lipids from LDL because of its ability to decrease hydroperoxides as well as HDL that reduce the accumulation of lipid peroxides in LDL,^5^ DM was associated with decreased HDL lipid and increased LDL. Therefore, the susceptibility to cardiovascular diseases (CVD) risk is about two to four fold higher in DM compared to healthy people. In fact, DM complications can lead to CVD, which is a major cause of morbidity and mortality worldwide.^23^

The decrease in PON1 enzyme activity reduces the ability of the body to normalize lipid-peroxidation, which thereby accelerates the oxidative stress. In diabetes there is overproduction of reactive oxygen species due to hyperglycemia or increased in free fatty acids and/or dyslipidemia.^24^ This issue has a prognostic indicator that the patients enrolled in the current study with uncontrolled glycemic status are expected to have a serious consequences of CVD due to low PON1 concentration.

There were significant association between PON1 and period of diabetes in T1DM and T2DM. This is supported by previous findings that document a strong association between duration of diabetes and elevated the risk of developing of CHD, as an independent risk factor.^25^ The results support the importance of PON1 as an early marker for the development of CVD among DM. Examining the role of genetic polymorphisms of PON1 in the Sudanese diabetes and how they might impact CHD complications among patients is strongly recommended. The current findings need to be confirmed in a larger study as the small the sample size is the major limitation of this study.

## CONCLUSION

Sudanese T1DM and T2DM have a lower PON1 concentration than healthy subjects, T1DM patients have lower level of PON1 than T2DM. PON1 was inversely correlated with bad lipids and duration of diabetes, but it has positive correlation with good lipids.
